# Older Women's Sexual Desire Problems: Biopsychosocial Factors Impacting Them and Barriers to Their Clinical Assessment

**DOI:** 10.1155/2014/107217

**Published:** 2014-06-05

**Authors:** Michelle Maciel, Luciana Laganà

**Affiliations:** ^1^Department of Clinical Psychology, Alliant International University San Diego, 10455 Pomerado Road, San Diego, CA 92131, USA; ^2^Department of Psychology, California State University Northridge, 18111 Nordhoff Street, Northridge, CA 91330, USA

## Abstract

Sexual desire is a major component of sexuality at any age, and inhibited desire is one of the main sexual dysfunctions reported by older women. Nonetheless, in medical settings, for a variety of reasons discussed herein, its assessment—as well as the assessment of older women's sexual health in general—is typically avoided or conducted by asking a single sex question. In this paper, we have reviewed the literature (most of which is preliminary in nature) regarding the main psychosocial and health factors that could impact older women's sexual desire, as well as potential obstacles to the assessment and treatment of this geriatric sexual issue. It is certainly advisable that medical care providers who are uncomfortable discussing older women's sexual concerns be prepared to make appropriate referrals to clinicians who possess the proper training to accurately assess and treat sexual challenges (and female sexual interest problems in particular) in this neglected patient population.

## 1. Introduction

Aging is a physiological, psychological, and social transition that typically affects sexuality. Continual sexual activity carries numerous health benefits throughout the life span: because sexual intimacy impacts sexual desire necessary for ongoing healthy sexual interactions in human relationships, problems in any area of the sexual experience should be addressed as part of a holistic health assessment (as discussed by Yee and Sundquist [1]). Sexuality and older women are issues, however, that are typically dichotomized rather than considered a naturally occurring combination to be explored and nurtured in their intersection. A seemingly perpetual belief is that sexual interest wanes considerably or completely with age [2]. On the contrary, the capacity to enjoy interactional sexual activity is not lost with the advancement of age, as sexual desire has been reported in 71% of women aged 80 to 102 who disclosed that they still fantasized or daydreamed about the opposite sex [3]. The empirical research highlighted below (spanning three decades) is reviewed herein to provide a broader framework of multiple aspects underlying reports of distress about and inhibition of an older woman's sexual desire. This was done in order to complement the knowledge of clinical practitioners who have been heavily influenced by the medicalization of sexuality in terms of physiological responses. This overt medicalization could lead health care professionals to use an exclusively functional diagnostic approach. It could also reinforce the notion of complete sexual equivalency between men and women as well as among all women (heterosexual, bisexual, lesbian, and transgender).

As we have noticed in our clinical practice, women do not usually define their sexual satisfaction or loss of desire based on the functioning of their sexual organs, but mostly based on the quality of the relationship within which sexual activity ensues. As a corollary, in this paper, we approached sexual desire issues in older age mainly through psychosocial explanations as we targeted the biopsychosocial factors impacting older women's sexual desire in particular, motivated by research finding such as those of Gefland [4], who reported that the most prevalent sexual issue in this population is indeed inhibited sexual desire (77%). Whereas this topic has inspired some anecdotal literature by several knowledgeable professionals in the medical and psychological fields, it has received relatively little empirical attention, which, in turn, impacted our ability to locate a larger body of pertinent empirical literature for this review. Investigating the prevalence and risk factors of sexual dysfunction in a nationally representative USA sample of individuals aged 57 to 85, Laumann and colleagues [5] reported the following prevalence rates for lack of interest in sex among older women: 45.4% (for age 57–64), 37.6% (for age 65–74), and 49.3% (for age 75 to 85). Thus, the prevalence of sexual desire issues seems to remain approximately the same for women once they reach the age of 55 (conversely, older men's reports of problematic orgasm and erection increased as they aged). Another noteworthy result was the lack of significant ethnic and racial differences for sexual interest in older age, suggesting that sexual desire and related concerns affect older women of all ages without racial exceptions. The authors' supplementary analyses revealed that the mechanism most likely linking older women's stressors and poor health to sexual issues such as low sexual desire is poor mental health, such as high levels of stress, anxiety, and depression (as discussed later in this paper). Conversely, an active sex life appears to provide many older people with a buffer against ill health in later life [6]. Because sexual activity has positive effects on older adults' physical health, it is important to resume a sexual relationship after an illness or traumatic event. Positive attitudes toward sexuality and the availability of a sensitive partner may facilitate the resumption of sexual interaction. In particular, Yee and Sundquist [1] listed five specific factors that appear to encourage sexuality (thus increasing sexual desire) in older women: a positive attitude towards sexuality, an active sex life in younger and middle years, good health, an interested and interesting partner, and the willingness to experiment sexually. Additionally, Persson [7] discovered that the following factors best predict engagement in sexual intercourse: (a) good mental health; (b) high levels of interest in sex among men when they were 20 years old; and (c) for women, having experienced premarital sex. Also important for continued sexual activity were cognitive flexibility and being able to adjust to the changes that accompany age (instead of battling against the inevitable), both of which allow the likelihood of emotional and sexual satisfaction, as well as sexual enhancement [8].

### 1.1. Objectives of this Review

Taking all the aforementioned factors into account, physicians, psychologists, nurses, and other health providers should not hesitate to raise the topic of sexual health and, in particular, sexual desire with older women patients. However, the assessment of the sexual concerns of older women is seldom done, for a variety of reasons succinctly covered herein. To shed some light on these neglected issues and to encourage reluctant clinicians to pay more attention to this area, we set as the main objective of this paper to inform medical professionals about biopsychosocial issues related to sexual desire among older women, with the secondary objective being to clarify reasons for the seldom assessment of sexual concerns in older female patients. In particular, in order to review the available literature on (1) factors impacting older women's lack of interest in sex and (2) reasons for clinicians' typical reluctance to assess geriatric sexual desire issues, we used the following major endpoints:

(for objective 1) physical/medical factors, psychological factors, demographic factors such as age, partner-specific factors, and social factors impacting sexual desire among older women;

(for objective 2) paucity of female health care providers, training and interdisciplinarity issues, and psychosocial factors impacting the limited assessment of sexual desire in older female patients. Once searches for sexual desire in particular yielded very few studies, we extended our searches to include the assessment of any sexuality issue pertinent to older female patients, thereby gathering a few additional studies.

### 1.2. Search Methods for Identification of Relevant Studies and Eligibility Criteria for Inclusion

We conducted electronic searches of Medline and PsychInfo (1968 to 2013) utilizing keyword search terms for our main and secondary goals. The two criteria for review inclusion of a study were (1) being written in English and (2) being pertinent to either one of our two goals. Selecting applicable studies that only utilized placebo or comparator control trials was not feasible, given the small body of the mostly cross-sectional literature obtained via our searches. For the same reasons, the type of literature that we were able to gather was not conducive to conducting a critical review. Indeed, most of the studies on either of our two goals were not only cross-sectional but also somewhat preliminary in nature, as they were not experimental and/or were mainly conducted on small groups of primarily Caucasian subjects. Given these challenging circumstances, we chose not to critique the scientific value of each study, focusing instead on offering the reader pertinent initial descriptive findings with direct clinical utility for either of our research goals.

Researchers interested in strengthening the evidence-based clinical applicability of the literature reviewed herein are encouraged to attempt to corroborate it in future research utilizing more rigorous methodology and more comprehensive as well as diverse samples (in terms of sexual orientation, disability status, and ethnic/racial/cultural diversity). The information provided below could be used as a stimulus for health care professionals' increased interest in covering sexual desire when assessing the overall health and quality of life of their older female patients. Clinical treatment issues concerning sexuality in older age have not been covered herein, to reasonably limit the scope of this review.

## 2. Studies on Biopsychosocial Factors Affecting Older Women's Sexual Desire

Sexual dysfunctions, as characterized by the Diagnostic and Statistical Manual of Mental Disorders (DSM-V, American Psychiatric Association [9]), reflect situational or generalized pain or disturbances in one or more psychophysiological processes that accompany the sexual response cycle and cause marked psychological and interpersonal distress. Until now, primary reliance has been afforded to the DSM-IV diagnostic criteria for sexual dysfunctions of the sexual response cycle, which encompasses the following four phases during which disorders may occur (at one or more of these phases): (1) desire: fantasies about sexual activity and the desire to engage in sexual activity; (2) excitement: a subjective sense of sexual pleasure and associated physiological changes—for women, the primary changes consist of vasocongestion in the pelvis, vaginal lubrication and expansion, as well as swelling of the external genitalia; (3) orgasm: a peaking of sexual pleasure with release of sexual tension and rhythmic contraction of the perineal muscles and reproductive organs—for women, contractions of the wall of the outer third of the vagina are experienced, although not always consciously as such, and the sphincter rhythmically contracts; (4) resolution: a sense of muscular relaxation and general well-being—for women, sexual response to additional stimulation may be immediate. Since the 1994 publication of the DSM-IV, research evidence has invalidated the linearity and precise division of phases in describing and treating sexual behavior for women in particular. For this reason, the 2013 publication of the DSM-V has sought to utilize an updated conceptualization of sexual behavior and to rectify and expand diagnoses and their respective criteria for sexual dysfunctions. One major change has been the merging of female hypoactive desire dysfunction and female arousal dysfunction into “female sexual interest/arousal disorder.”

According to Levine's conceptualization of sexual desire [10], the latter is comprised of three related elements: (1) drive, (2) expectations/beliefs/values, and (3) psychological motivation. Within this framework, drive is conceptualized as biologically based and experienced as spontaneous interest typically through genital tingling, sexual thoughts or fantasies, and increased sexual interest in others nearby. Moreover, testosterone is known as necessary for sexual desire, which declines in both men and women with age. Occurring cognitively, expectations, beliefs, and values affect the interest in behaving sexually. Last, psychological motivation is defined as a willingness or unwillingness to behave sexually with a partner. Also, according to Levine, an important feature in understanding sexuality (and sexual desire in particular) is sexual equilibrium, which has an interpersonal nature and is characterized by a balance in sexual capacities and perceptions of those capabilities between two people. These capacities include capability for desire, arousal, and orgasm, which are highly responsive to psychosocial forces. Healthy sexuality enhances patients' psychological well-being and, at the same time, psychological well-being enhances healthy sexuality. In contrast, disequilibrium occurs when there is dissatisfaction in one or both partners with a nonsexual relationship, or when sexual relations occur less than ten times per year. Disequilibrium could become a significant medical and/or mental health issue. A number of medical and psychosocial factors listed below could play a key role in women's low sexual desire.

### 2.1. Menopause and Other Physical Causes

Not all women experience a negative impact on sexual health as a result of menopause; McCoy and Davidson [11] found that the older women in their samples reported no major loss of health and sexuality. For many women, however, changes in hormonal levels during and after menopause result in varied changes in the genitourinary system. Testosterone deficiency and decreased secretion of estrogen may result in vaginal dryness and painful intercourse, atrophic skin changes, shrinkage and atrophy of the clitoris and vagina, diminished sensation, urogenital prolapse, and urinary incontinence. Other empirical results documented in the literature include reduction in skin's sensitivity to touch, reduced olfactory epithelium's sensitivity to smell, loss of muscle bulk and bone mass, loss of sense of well-being, cognitive slowing, and loss of concentration [1, 12]. Researchers have reported that hormone replacement therapy (HRT) could improve quality of life for some women [13]. Compared to placebo administration, use of synthetic estrogen treatment may reduce some of the physical symptoms of menopause, yet it does not significantly ameliorate depression or overall quality of life [14]. In consideration of the increased risk for cancer, stroke, and blood clots related to the use of synthetic hormones, research within the past decade has shown that long-term use of such hormones is inadvisable for some women [15]. Some scholars have reported that, although randomized control trials are lacking, the findings of several clinical outcome studies indicate that bioidentical hormones are related to lower risks of breast cancer and cardiovascular disease and are also more effective than synthetic or animal-derived hormones (e.g., Holtorf [16]). Understandably, conflicting and/or inconclusive findings in this area could lead to many practitioners being confused regarding the kind of hormonal treatment that would be optimal for a symptomatic aging woman with low hormonal levels who is reporting sexual interest problems. To further complicate the clinical picture, in addition to hormonal imbalances, a number of other physical factors could alter sexual desire, including neurologic, vascular, or other conditions associated with illness/surgery or medications [17].

### 2.2. Body Image and Self-Worth

Butler and Lewis [18] noted that older women are typically viewed as inactive, unhealthy, asexual, and ineffective in society. Older women's social contexts and sexual norms are likely to impact their sexual desire by affecting the way they feel about their bodies, appearance, and sexuality. The resulting pejorative self-views and expectations could emphasize any reduced cognitive behavior, increase depression, and reduce sexual interest and activities. Inevitably, the human body changes in its biology and its appearance over time, but a woman who perceives the aging process as a positive reflection of her maturity and self-confidence could even experience enhancement of her desirability and sexual desire. Conversely, there is literature suggesting significant correlations among a woman's perceptions of the physical signs of aging as unattractive, poor body image, and loss of femininity [19]. Such self-perceptions could result in a decrease in sexual desire, as sexual activity requires emphasis on the body, which could become a source of anxiety and depression for women who are not successful at coping with their bodily changes. Some of them choose to use cosmetic surgery and other image enhancers to preserve their youthful looks, but, at times, these attempts can result in somewhat uneven to even grotesque results that are doubtfully effective at enhancing self-esteem. Within the past few years, a preoccupation with cosmetic genitoplasty has been noted, mainly to address labial reduction and vaginal tightening. However, according to researchers such as Lih and Creighton [20], genital surgery is risky, does not empower women to resolve body image issues, and often leads to a preoccupation with the next unattractive physical attribute to be altered. If done safely from a medical viewpoint, with attentiveness to potential short- and long-term side effects, cosmetic treatments and medical procedures that can enhance aging women' self-image and corresponding confidence in their sex appeal—potentially boosting their sexual desire without major risks—are certainly an option.

### 2.3. Quality of Older Women's Intimate Relations and Age in Relation to Sexual Desire

While menopause is undoubtedly culturally experienced and defined, Western industrialized societies continue the tradition of medicalization in the approach to this life transition for a woman. At the same time, some research attention is being focused on multidimensional postmenopausal sexuality, with the resurgence of qualitative methods of inquiry that place priority on the context of the relationship in which sexuality occurs (e.g., Laganà and Maciel [21]). Within a social context, the association between women's quality of their intimate relationship and sexual functioning is fundamental to better addressing emotional and sexual challenges. A person's age is not a reliable predictor of type and quality of intimate relationships, and emotional intimacy typically remains a significant need regardless of age. Research shows that the healthy couple is close yet has autonomous and differentiated identities [22], and the corresponding sexual-developmental task is to sustain pleasure [10]. However, the empirical literature suggests that intimacy may not promote sexual desire [8], whereas mystery, freshness, and risk are typically necessary to elevate components of passion that may be absent in these relationships.

### 2.4. Erectile Disorder in the Sexual Partner and Lack of a Sexual Partner

Older couples are at a greater risk of becoming asexual; therefore, they must devote considerable energy toward keeping intimacy alive and healthy. Contrary to the misperception that women are usually in control of determining whether an older heterosexual couple ceases sexual activity, sexual desire more commonly declines among men, usually due to erectile dysfunction (ED) [23]. The stereotype of the asexual menopausal woman could originate mainly from the sexual functioning problems of aging men, which are not conducive to older women's expression of sexual interest. Feldman and colleagues [24] found that the majority of women in their sample were more likely to be concerned about their partner's sexual functioning, as 67% of men experience some degree of ED by age 70. Research shows that most women and men report that ED is a major reason for decreased sexual activity [25]. Although very popular these days, Sildenafil and similar medications have side effects and do not always work for ED. Empirical evidence suggests that the most preferred intimate activity among sexually active men and women aged 80 to 102 is mutual caressing, followed by masturbation and penetrative sex [3]. Moreover, one of the most significant relational changes occurring in older age for many heterosexual women is that they will likely outlive their male partners. In a study of USA women over 60 years old by Diokno and colleagues [26], 55.8% of married women were sexually active compared with 5.3% of unmarried women. Almost half of the sample of Hispanic-American older women recruited in Laganà and Maciel's [21] study reported having sexual fantasies, yet many of these women had no desire to engage in sexual activity due, at least in part, to unavailability of an appropriate partner. Many women who would otherwise remain sexually active into older age are forced into sexual abstinence due to lack of a partner or access to an intimate companion. Malatesta and colleagues [27] reported that, for women without a sexual partner, sensual interactions are more important than explicitly sexual experiences; thus, nonsexual intimacy, including emotional and cognitive intimacy, may serve a major potential role in satisfying older women's need for intimacy.

### 2.5. Religiosity, Social Context, and Social Norms about Sexuality

Back in 1978 (yet still pertinent now), Rubenstein [28] reported that factors affecting the sexual activities of older adults include shame, sin, and other religious and cultural aspects. Growing religious devoutness could explain why a woman's interest in sex might decline with age [21]. While more modern interpretations of religions are not necessarily averse to a loving sexual relation outside of procreation, and shame may be more indicative of negative perceptions of sexuality, older adults and believers typically are less sexually permissive than young people and nonbelievers, regardless of educational level [29]. Concerning social context, lack of privacy, especially if the older woman lives with her family or in a geriatric facility, may further limit her opportunities to be sexual, and social pressure could steer her towards celibacy. As to social norms, Riley [30] reported that older women face more cultural obstacles related to roles prescribed by societal values and norms than older men. As described in classic literature from the 1970s that still applies today, cultural traditions and restrictions may influence people's perspectives that sex for women is unacceptable, dangerous, taboo, or inevitably to be completed by a certain chronological date [31]. Moreover, prejudice, misapprehension, and misinformation in the older population are a significant source of intense feelings of derision, denial, and despair about sex [32]. Furthermore, in a literature review, Guan [23] pointed out that Masters and Johnson, back in 1968 [33], insightfully acknowledged that aging itself is not what they observed to be the cause of cessation of sexual activity in older patients, but that sexual norms correlate highly with sexual activity and usually discourage sex in older age. Not much seems to have changed now compared to the attitudes held toward older women's sexuality in the past decades; thus, it is not surprising that the topic of their sexual desire is not receiving enough clinical or research attention.

## 3. Research Pertinent to Potential Barriers to Older Women's Sexual Health Assessment

Typically, older men have an advantage over older women as it pertains to assessing their sexual problems. Sobecki and colleagues [34] highlighted that men are counseled more than women about the impact of medical treatment on sexual functioning as part of the decision-making process regarding their need to adhere to a particular medication regimen. According to the aforementioned authors, health care physicians feel more comfortable talking about sex with men simply due to the availability of FDA-approved erectile dysfunction drugs designed for them. Moreover, Feldhaus-Dahir [35] noted that the smaller success rate of Sildenafil trials for women compared to the results achieved on men has adversely influenced the medical community in addressing women's sexual health. Without a magic pill to alleviate biological symptomatology, many distinguishable psychosocial sexual concerns of older woman have frequently gone undetected by physicians. Some researchers have discovered that older women are indeed interested in discussing their sexual concerns with their physicians (e.g., Andrews [36]), but, as emphasized in the literature, medical professionals have an inconsistent and usually avoidant approach to this topic [35]. Consequently, the neglect of the assessment of sexual concerns, typically encountered in the hospital visit, virtually places diagnostic responsibility on the older patient, who is expected to raise likely embarrassing sexual questions. This is hardly an ideal situation for an older woman in need of help with her sexual problems. The denial of older women's sexuality is further amplified by glaring factors such as the antiseptic aesthetic of the uniformed medical environment, making feeling comfortable bringing up sexual problems even more challenging for older women. As succinctly described next, health care professionals' ability to address women's sexual concerns might be unintentionally constrained, stemming from a variety of reasons, including the limited availability of female physicians who are interested in discussing older women's sexuality, the limited training in the assessment of the sexual dysfunctions of older patients, and some specific characteristics of health care providers that could contribute to older women patients' reticence about bringing up sex.

### 3.1. Few Female Health Care Providers Are Assessing the Sexual Concerns of Older Women

Nusbaum and colleagues [2], upon conducting a cross-sectional survey of women seeking routine gynecological care, found that those aged 65 and older (17%) had just as many sexual concerns as younger women did, with most of them indicating that they would discuss these concerns if their physician candidly raised the topic (97%). Additionally, 58% to 73% of the older women in the sample reported being less embarrassed about raising sexual issues with their physician if this person had the following characteristics: (a) professional demeanor; (b) comfort with the topic; and (c) a kind, understanding, and empathic disposition. Research by Sobecki and colleagues [34] shows that nearly two-thirds of obstetrician-gynecologists (OB-GYN) in their sample routinely ask about patients' sexual activity, whereas only 40% routinely inquire about sexual concerns or dysfunctions, and just 29% ask about sexual satisfaction. Female physicians under the age of 60 are more likely to address sexual activity, orientation, or identity with female patients (and gynecologists in general are more likely to screen for sexual dysfunction than other physicians). Other researchers have pointed out that the most likely place to address older women's concerns about sexuality is during the medical appointment with their female clinicians (e.g., Gott and Hinchliff [37]). Yet, when their doctors do not ask, these patients could assume that their sexual problems are not a viable topic for discussion; as a result, they could feel anxious about initiating the conversation and, thus, their clinical needs for help in this area could go unmet. Perhaps having a same-sex provider could help reduce embarrassment on both parts. Although we were unable to locate literature on the actual rates of female gynecologists/physicians who are routinely assessing the sexual needs of older women, the available empirical evidence points to the need for more female medical professionals to be able to conduct in-depth evaluations of these patients' neglected sexual concerns.

### 3.2. Training Deficits and Lack of Interdisciplinary Mindset in Some Medical Treatment Settings

When studying the professional well-being of health care providers, the current paper's first author [38] underscored the strong association between relevant training opportunities and a sense of confidence and competence in practice; this applies to a clinician's level of comfort in addressing sexual topics when assessing older patients. Pauls and colleagues [39] investigated the perception of quality of training received for the assessment of women's sexual dysfunction among physicians who are members of the American Urogynecologic Society. Fifty percent of the respondents who received postresidency training in urogynecology reported that their training in female sexual dysfunction was unsatisfactory. All respondents believed that an appropriate screener for female sexual problems was just one question about sexual activity during a history intake; moreover, only 22% of urogynecologists reported asking this question. It is alarming that a single question on being sexually active (without assessing desire, orgasm, genital pain, or other related topics) would be considered sufficient when assessing older women's sexual functioning. Yet, even this one question is usually not posed in a medical setting to start with. Feldhause-Dahir [35] emphasized the need for contemporary adjustments to training curricula and practicum experiences, as inadequate clinical communication habits during training generally carry over into the professional setting, likely hindering physicians' ability to address sensitive topics such as sexual health in older age.

### 3.3. Non-Training-Specific Reasons for the Limited Assessment of the Sexual Problems of Older Women

The mission of comprehensive health treatment obliges physicians, psychologists, and other health care providers to place importance on the sexuality of their patients. Yet, the reasons for avoidance of this topic among many health care professionals are multifaceted. First, reproductive biology and the strongly male-gendered evolutionary perspective have likely influenced medical viewpoints, typically rendering women's sexual functioning unimportant beyond the reproductive years. Researchers have pointed out that, as detrimental sexual stereotypes surrounding the older woman persist, others expect her to be incapable of having sex, to be sexually undesirable, and to not desire sex [40]. Regrettably, ageism may produce feelings of embarrassment, shame, and anxiety for older women that prevent them from discussing sexual concerns. Empirical findings show that, while the frequency of a woman's sexual activity diminishes with advanced age, her sexual interest and ability, however, generally do not [41]. Ageist attitudes regarding sexuality were detected in a study by Gott and colleagues [42], who found that the general practitioners from their UK sample did not proactively address the sexual health of older people and deemed this issue an illegitimate topic for discussion (likely based on stereotypic views of sexuality and aging rather than on individual patient experiences). As reported by Gott and Hinchliff [37], older adults stated that their general practitioners did not provide information about sexual issues or discuss the risks and side effects of their medical condition and its associated pharmacotherapy (although the latter could directly impact their sexual functioning). Likewise, Pauls and colleagues [39] discovered that 20% of urogynecologists from their sample did not even attempt to assess female sexual problems.

In a study on senior medical students, Merrill and colleagues [43] found that the traits of shyness and social anxiety predicted the likelihood of embarrassment of these students in taking a patient's sexual history. A low level of empathy for patients' psychosocial problems was also associated with the belief that an individual's sex history is unrelated to the conceptualization of a patient's problems. Medical students' low self-esteem was associated with the belief of not being adequately trained in taking a sexual history. Trainees who held this strong belief also had a high degree of authoritarian views and homophobia, which, in turn, could preclude assessing whether homosexuality is a life-long choice for the older female patient or is adopted as a reaction to the scarcity of male partners in older age (we found no empirical evidence on this potentially controversial yet clinically relevant topic). The authors also identified three reasons why medical doctors usually fail to take adequate sexual histories, namely, personal embarrassment about directly discussing sex with patients (25%), the conviction that the sex history is insignificant to the patient's principal complaint (93%), and the belief that they received insufficient training in taking a sexual health history (50%, a topic already discussed in Section 3.2.).

## 4. Suggestions for Doctor-Older Female Patient Communication and for Future Research

As gleaned from the aforementioned literature, a physician may be more inclined to address the topic of sexual health with women patients when this health care professional is a woman, a gynecologist, under the age of 60, more socially empathic, less homophobic, sufficiently trained in female sexual health and in taking a sexual history, and oriented toward a biopsychosocial conceptualization of patient complaints. Realistically, this combination of traits and training is hardly common. Yet, according to Elias and Sherris [44], the expansion of services for older women places new demands on multidisciplinary sexual health teams to receive preservice and refresher training in order to (a) enhance their attitudes toward older women, (b) understand how to interact with these patients in nonpatronizing and culturally appropriate ways, (c) promptly identify and treat complex health problems in older women that could be affecting their sexual functioning, and (d) investigate whether current medications might be affecting these patients' sexuality adversely.

We believe that the use of sexual functioning questionnaires, as opposed to direct interview methods or at least in addition to those methods, may be useful in initiating discussions of sexual concerns with older women. If, after sufficient training and practice, a physician continues having difficulties with the topic of sexual health, he/she could refer this patient out to a psychologist or other clinician specializing in psychosexual functioning. To say the least, just bringing up the topic of sex and asking an older woman patient whether she would like to discuss her sexual health concerns with a specialist would be helpful. Then, the underpinnings of her sexual problems could be addressed, ideally using appropriate interdisciplinary treatment plans (which are outside the scope of this review). A physician would likely benefit from exploring the origin of his or her discomfort with the topic of sex in aging patients, as she or he could learn to overcome this difficulty, which could enhance a sense of professional competence and help older patients who could be struggling with unspoken, sensitive sexual concerns.

There is still much to be investigated more in-depth, or even for the first time, in the area of older women's sexual desire problems in particular and sexual functioning in general. For instance, interested researchers should focus on the sexual health of nonmainstream groups, such as older women with nontraditional sexual orientations or those living with different kinds of physical limitations and disabilities. Also, little empirical evidence is published on the relationship between ethnicity and the sexual health of older women (as pointed out by Vincent [6]). Unfortunately, diversity of any kind has been neglected in the investigation of older women's sexual desire and sexual functioning in general. Furthermore, many training questions are currently unanswered, including how to best train physicians—those who are not willing to refer older women out for the assessment and treatment of sexual problems as well as physicians who are interested in improving their clinical expertise on sexual health—for the conduction of reliable and comprehensive assessment of sexual functioning in older women. In view of the extensive aging of the current population, this topic should be addressed more adequately as soon as possible, by interested physicians as well as by individuals in charge of medical school training and continuing education curricula.

## 5. Conclusive Comments

In this review, we have succinctly covered biopsychosocial factors impacting sexual desire among older women as our primary objective, while, as a secondary (nonetheless clinically important) objective, we have presented available evidence on potential reasons for the typical avoidance of the assessment of older women's sexual health in general, and sexual desire in particular, within medical/clinical settings. As reported by Lindau and colleagues [45], although older women state that they have multiple sexual concerns such as a lack of sexual desire, these problems are seldom discussed with their physicians, who, as suggested by the aforementioned authors, should make an effort to become involved in their patients' education and counseling regarding sexual problems. Main deterrents to initiating geriatric clinical research in this area could be the anticipated difficulties inherent in soliciting information of a very delicate sexual nature from older women. Some of the research findings summarized above, however, suggest that these women are interested in discussing their sexual concerns with their health providers. Furthermore, Walker and Ephross [46] reported that 80% of older adults living in long-term care facilities or attending a senior center agreed that masturbation is an acceptable sexual activity for older women; this research outcome speaks to the fact that older adults are well aware of the existence of older women's sexual desire, a topic that is generally taboo in society and in medical settings. Such an empirical finding is also indicative of the general open-mindedness of older adults regarding the fact that older women's sexual needs should be fulfilled. Yet, empirical data on masturbation among older women is very scarce and, when available, is indicative of a widespread nonengagement in this sexual activity: in a 2012 study on older adults living in Spain, Palacios-Ceña and colleagues [47] reported a very low prevalence of older women's masturbation within the preceding year (11.7% among women aged 65 to 74 and only 5% among women aged 75 and older). More research on this taboo topic is definitely needed to clarify reasons for these low statistics.

Low sexual desire, as elucidated in this review and emphatically pointed out by researchers such as Miner and colleagues [17], is often untreated due to the embarrassment of clinicians and their patients to introduce the topic within clinical settings. The aforementioned authors encouraged health providers to utilize short, validated questionnaires to assess inhibited sexual desire, including the Decreased Sexual Desire Screener, which can be completed in the waiting room and has a very high reported sensitivity (of 0.946 with a North American sample and of 0.960 with a European sample) [48]. Using such a tool might provide an opener for the discussion of sexual concerns between patients and health care professionals. Moreover, conducting and disseminating additional research in this area would have many benefits. The latter include fostering the facilitation of health care policy advancements for older women, encouraging more appropriate referrals by physicians to professionals who possess sexual health training, as well as allowing the deconstruction of the various myths that have been perpetuated through the past decades about older women's alledged asexuality. Among all physicians, OB-GYNs appear to be best positioned to address older women's sexual concerns-although just checking off yes or no boxes on a list of sexual activities is not likely to shed enough light on changes in quality of sexual functioning as women grow older. Overlooking older women's sexuality can directly and negatively affect policies and the provision of high-quality care in the area of geriatric sexual health. We are hopeful that this review has sparked some interest in finding ways to successfully address the current neglect of older women's sexual desire in particular and sexual health in general.
